# Cognitive deficits in adult m.3243A>G‐ and m.8344A>G‐related mitochondrial disease: importance of correcting for baseline intellectual ability

**DOI:** 10.1002/acn3.736

**Published:** 2019-03-27

**Authors:** Heather L. Moore, Thomas Kelly, Alexandra Bright, Robert H. Field, Andrew M. Schaefer, Alasdair P. Blain, Robert W. Taylor, Robert McFarland, Doug M. Turnbull, Gráinne S. Gorman

**Affiliations:** ^1^ Wellcome Centre for Mitochondrial Research Institute of Neuroscience Newcastle University Newcastle upon Tyne NE2 4HH United Kingdom; ^2^ Institute of Health & Society Newcastle University 3rd Floor, Sir James Spence Institute Royal Victoria Infirmary Queen Victoria Road Newcastle upon Tyne NE1 4LP United Kingdom; ^3^ Department of Neuropsychology Royal Victoria Infirmary The Newcastle upon Tyne Hospitals NHS Foundation Trust Newcastle upon Tyne NE1 4LP United Kingdom

## Abstract

**Objective:**

To determine the cognitive profile of adult patients with mitochondrial disease, and the effect of disease severity on cognition.

**Methods:**

Using a prospective case‐control design, we compared cognition of patients to normative data and to matched controls, assessed three times over 18 months. Forty‐nine patients with m.3243A>G (*N* = 36) and m.8344A>G (*N* = 13) mtDNA mutations and 32 controls, matched by age (±5 years) and premorbid cognition (±10 WTAR FSIQ points), participated. Participants completed neuropsychological assessments of general cognition (WAIS‐IV), executive function (D‐KEFS), and memory (WMS‐IV). Potential predictors of cognition were explored.

**Results:**

Patients show mild‐to‐moderate premorbid cognitive impairment, but substantial impairment in current general cognition and distinct domains, including verbal comprehension, perceptual reasoning, working memory, processing speed, and memory retrieval. Executive dysfunction may be caused by slower decision‐making. Patients performed worse than controls, except on memory tasks, indicating intact memory, when premorbid cognition is controlled for. Premorbid cognition and disease severity were consistent predictors of cognition in patients; however, cognitive decline appears slow and is unlikely in the short‐term, when other disease‐specific factors remain stable.

**Interpretation:**

Patients should be monitored to facilitate early identification of a complex profile of cognitive deficits and individuals with higher disease burden should be followed up more closely. On development of cognitive difficulties, appropriate compensatory strategies should be determined through in‐depth assessment. Using strategies such as slower presentation of information, multiple modes of presentation, active discussion to aid understanding and decision‐making, and use of memory aids, may ameliorate difficulties.

## Introduction

Mitochondrial diseases are a common group of genetic neuromuscular disorders characterized by genotypic and phenotypic heterogeneity with a prevalence comparable to many other genetically determined neurodegenerative diseases.^1^ Neurological features in adult patients with mitochondrial disease are prominent and include ataxia, seizures, stroke‐like episodes, migraine, psychiatric features, and encephalopathy. Whilst there is significant clinical variability, neurological impairment remains one of the hallmarks of mitochondrial disease and cognitive impairment, currently, is one of the least understood aspects.

In patients with mitochondrial disease, high rates of cognitive delay, cognitive and memory impairment, and dementia have been reported in small case studies and review articles only. However, these studies are primarily a retrospective analysis of patients manifesting high genotypic variability and disease burden.

Focal cognitive deficits of memory, executive functioning, visuospatial abilities, and processing speed have been reported in small‐scale, systematic investigations carried out at a single time point, with marked variability in results.^2^, ^3^, ^4^, ^5^, ^6^, ^7^, ^8^, ^9^, ^10^, ^11^ While studies have suggested mtDNA genotype‐specific differences in cognition^6^; group sizes have been insufficient to identify specific profiles and cognitive performance may better relate to disease burden.^11^

We sought to evaluate cognitive ability and delineate potential predictors of cognition in patients with the m.3243A>G and m.8344A>G‐related mitochondrial diseases^1^ compared to standardized normative data^12^, ^13^, ^14^ and to age‐ and premorbid cognitive ability‐matched control participants over time.

## Methods

### Study design

**Figure 1:**
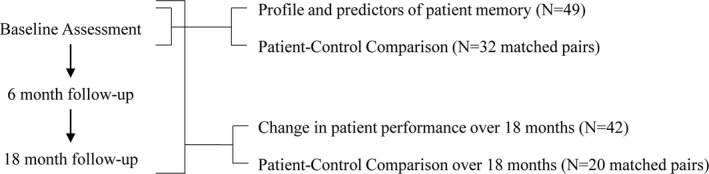
Participant numbers for each sub‐analysis of study.

We performed a prospective case‐control study comparing cognition in patients with mitochondrial disease to the norm and to age‐ and premorbid cognitive ability‐matched controls, assessed three times over 18 months (Fig. 1). Predictors of cognition were also explored. This study was approved and performed under the ethical guidelines issued by our institution and complied with the declaration of Helsinki. All participants gave written informed consent prior to study inclusion.

### Participants

**Figure 2:**
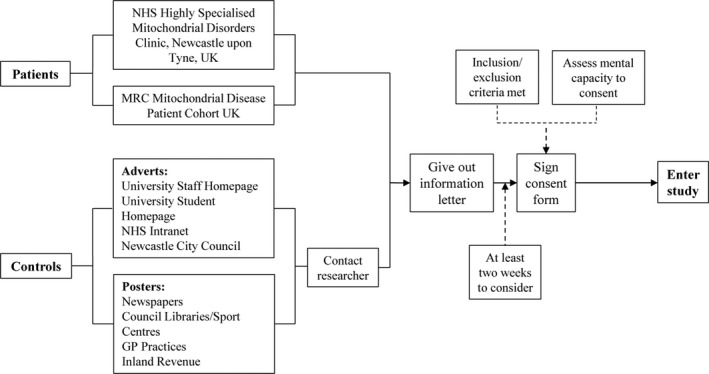
Recruitment and consent process for patients mitochondrial disease and control participants.

Forty‐nine adult patients with mitochondrial disease, with either m.3243A>G (*N* = 36) or m.8344A>G (*N* = 13)–related mitochondrial disease, and 32 controls matched for age (±5 years) and premorbid cognitive ability (±10 Wechsler Test of Adult Reading^15^ (WTAR) FSIQ points) participated. Matching criteria were designed to elucidate cognitive change associated with mitochondrial disease, unrelated to age or premorbid cognitive functioning. Figure 1 shows participant numbers for each sub‐analysis. Recruitment and consent process for patients with mitochondrial disease and controls is outlined in Figure 2.

Patient scores were compared to population‐based norms from the WTAR, WAIS‐IV, D‐KEFS, and WMS‐IV data manuals (Data S1).

### Apparatus

Each participant completed a battery of tests assessing premorbid cognitive ability, general cognitive ability, verbal comprehension, perceptual reasoning, working memory, processing speed, executive function, and memory, using the WTAR,^15^ WAIS‐IV,^13^ D‐KEFS,^12^ and WMS‐IV.^14^ Table 1 gives detailed information about cognitive assessments used and the domains that they map on to. Cognitive data were translated from raw scores into population‐based standard scores using normative data^12^, ^13^, ^14^, ^15^; WTAR and WAIS‐IV have a normative mean of 100 (SD = 15); D‐KEFS and WMS‐IV have a normative mean of 10 (SD = 3), except WMS‐IV recognition tasks, which were translated into percentiles.

**Table 1:** Cognitive assessments used, domain of cognitive functioning that they map on to, and definition of that domain

Test	Subtest	Acronym	Domain of functioning	Definition/Real World Context
Wechsler adult intelligence scale‐IV (WAIS‐IV)^13^	‐Full Scale IQ	FSIQ	General cognitive functioning	Average intellectual ability across a range of domains and skills.
	‐Verbal Comprehension Index	VCI	Verbal comprehension	Ability to use words and language to solve problems.e.g. understanding more complex language (e.g. in a consultation).
	‐Perceptual Reasoning Index	PRI	Visuospatial and perceptual reasoning	Use of visual information to understand and interact with the environment.e.g. finding your way around a car park, putting together flat pack furniture.
	‐Processing Speed Index	PSI	Processing speed	How quickly a person can take information in and use it.
Delis‐Kaplan executive function system (D‐KEFS)^12^	‐Verbal Fluency	VF	Verbal executive function tapping working memory, self‐monitoring, inhibition, task switching, and cognitive flexibility	The control processes that guide behavior, help you to plan what to do, monitor whether a plan is working, change plans if the first isn't working, stop yourself from doing something that you shouldn't.
	‐Tower		Nonverbal executive function utilizing planning, rule learning, and inhibition	
Wechsler adult memory scale‐IV (WMS‐IV)^14^	‐Logical Memory	LM	Immediate, delayed, and recognition memory for narrative content	Ability to learn, remember, and recall verbal information.e.g. remembering a shopping list, a conversation with a friend, family member, or healthcare professional.
	‐Verbal Paired Associates	VPA	Immediate, delayed, and recognition memory for word pairs when providing a cue, and free recall (without a cue)	
Birt Memory and Information Processing Speed Battery (BMIPB)^35^			Motor speed	How quickly a person can physically complete a simple task that does not require any thinking. This will impact performance on any timed task.

WTAR FSIQ,^15^ number of years in education, Newcastle Mitochondrial Disease Adult Scale (NMDAS),^16^ mtDNA genotype, anticonvulsant medication, medication for depression, Beck's Depression Inventory (BDI),^17^ Beck's Anxiety Inventory (BAI),^18^ % mtDNA mutation level in urine,^19^ age, and gender were considered as predictors of cognition in patients with mitochondrial disease. Items regarding seizures, mood, and cognition were excluded from the NMDAS score to prevent overlap in variance with other predictors.

### Procedure

WMS‐IV immediate memory was assessed, followed by delayed memory and recognition memory (and Free Recall memory in the case of VPA) after a 20–30‐min interval, in which WAIS‐IV and D‐KEFS subtests were administered. Participants also completed the WTAR, BDI, and BAI. Cognition was assessed three times over 18 months (baseline, 6 months, and 18 months). At each assessment, patients with mitochondrial disease underwent physical examination by experienced clinicians (GSG, AMS, RMF and DMT) using the NMDAS.

### Statistical analyses

Participants were compared to normative data using *Z*‐tests, executed in Minitab Version 16.^20^ They were compared to matched control participants at baseline and over time, using paired samples *t*‐test, Wilcoxon signed ranks test for paired samples, and repeated measures ANOVA, performed in SPSS Version 19.^21^ Linear regression was executed in SPSS to determine predictors of cognition in patients with mitochondrial disease at baseline assessment. Data S1 gives a full description of statistical analyses. To correct for multiple statistical analyses on different cognitive subtests, *P*‐values were adjusted using the Holm method,^22^ which is more powerful than the commonly used Bonferroni correction.

## Results

Table 2 shows the baseline characteristics of patients with mitochondrial disease and controls. Number of years in education at baseline assessment differed between the groups (*Z*(32) = −2.04, *P* = 0.042).

**Table 2:** Baseline characteristics of participants at each time point

	Patient characteristics		Baseline patient‐control comparison		18 month patient‐control comparison	
	Baseline	Patients Completing Study	Patient	Control	Patient	Control
N	49	42	32	32	20	20
Age (years)	46.24 (12.15)	46.76 (11.21)	47.19 (13.59)	47.25 (14.42)	43.85 (12.29)	43.35 (12.53)
Gender						
Male	23 (46.94%)	19 (45.24%)	14 (43.75%)	14 (43.75%)	10 (50%)	11 (55%)
Female	26 (53.06%)	23 (54.76%)	18 (56.25%)	18 (56.25%)	10 (50%)	9 (45%)
mtDNA genotype						
m.3243A>G	36 (73.47%)	33 (78.57%)	24 (75%)	\	17 (85%)	\
m.8344A>G	13 (26.53%)	9 (21.43%)	8 (25%)	\	3 (15%)	\
% mtDNA mutation level in urine	60.87% (26.47)	57.52% (26.44)	58.36% (27.08)	\	56.76% (29.33)	\
No. of years in education	13.67 (2.58)	13.62 (2.64)	14.41 (2.83)	15.72 (2.76)	14.95 (2.61)	16.20 (2.83)
WTAR	93.45 (12.59)	93.17 (12.87)	100.31 (8.87)	101.12 (8.58)	102.10 (8.77)	103.25 (7.95)

N.B. Values provided for point of study entry. Data are Mean (SD) or *N* (%).

### Patients compared to normative population data at baseline assessment

Table S1 shows summary data for comparison of patients to normative data on cognitive assessments.

#### Premorbid cognition

Patients performed significantly lower than the normative population on the WTAR, with a pattern suggesting mild (1SD) to moderate (1.5SD) rather than severe (2SD) premorbid cognitive impairment (1SD = 27%; 1.5SD = 18%; 2SD = 0%).

#### General cognitive functioning

Patients scored significantly lower than the normative population on the WAIS‐IV FSIQ, with high rates of impairment at both 1SD (45%) and 2SD (22%), showing clear evidence for current cognitive impairment. This was reflected in significantly worse scores on all WAIS‐IV subtests. PRI performance was significantly better than PSI (*F*(2.59) = 4.86, *P* = 0.005; Greenhouse‐Geisser correction; PRI‐PSI: *P* = 0.008).

#### Executive function

Patients performed significantly lower than the normative population on VF subtests, with higher than expected rates of impairment at 1SD (Letter = 31%; Category = 49%, Switching Correct = 43%, Switching Accuracy = 27%) and 2SD cut‐offs (Letter = 10%; Category = 7%, Switching Correct = 20%, Switching Accuracy = 6%). VF Category and VF Switching Correct did not differ significantly (*t*(48) = −1.92, *P* = 0.061, *d* = −0.55), indicating normal VF Switching performance, when taking account of semantic fluency.

On the D‐KEFS Tower test, patients scored significantly worse than the norm on all subtests but the Tower Move Accuracy Ratio. There were low to normal rates of impairment at 1SD (Total: 16%; Time Per Move Ratio: 33%; Move Accuracy Ratio: 6%; Rule Violations Per Item Ratio: 12%) and 2SD (Total = 6%; Time Per Move Ratio = 25%; Move Accuracy Ratio = 2%; Rule Violations Per Item Ratio = 4%) for all but Time Per Move Ratio; therefore, a small number of more severely affected participants may have skewed the results.

#### Memory

Patients scored significantly lower than the normative population on LM I and LM II, with higher than expected rates of impairment at 1SD (LM I = 42.86%; LM II = 30.61%) and 2SD (LM I = 14.29%; LM II = 12.24%). Patients demonstrated poorer free recall than expected in relation to recognition memory (*P* < 0.001). Conversely, they performed similarly to the norm on VPA I and VPA II and patients showed similar performance on prompted recall versus recognition (*P* = 0.096). VPA Free Recall performance was significantly worse than the norm, with higher rates of impairment at 1SD (*M* = 30.61%) and 2SD (*M* = 10.20%), replicating the LM free recall impairment.

### Patients compared to matched controls at baseline assessment

#### General cognitive functioning

Patients achieved significantly worse WAIS‐IV FSIQ and Index scores than controls (FSIQ: *t*(31) = −6.47, *P* = 0.005, *d* = −2.32; VCI: *t*(31) = −5.96, *P* = 0.005, *d* = −2.14; PRI: *Z* = −3.25, *P* = 0.005, *r* = −0.41; WMI: *t*(31) = −3.52, *P* = 0.005, *d* = −1.26; PSI: *Z* = −4.14, *P* = 0.005, *r* = −0.52; Table S2B). Including BMIPB Motor Speed in a mixed model ANCOVA identified a significant interaction between motor speed and disease status (*t*(29) = −2.18, *P* = 0.038, *d* = −0.78); patients with faster motor speed did not perform worse than matched controls, whereas patients with slower motor speed did show PSI decrements (Figure S1).

#### Executive function

Patients performed significantly worse than controls on VF Letter (*Z* = −3.33, *P* = 0.004, *r* = −0.42), VF Category (*t*(31) = −4.76, *P* = 0.004, *d* = −1.71), VF Switching Correct (*t*(31) = −3.89, *P* = 0.004, *d* = −1.40), and VF Switching Accuracy (*t*(31) = −4.09, *P* = 0.004, *d* = ‐1.47; Table S2B). The mean difference on each VF subtest was around 3 IQ points (Table S2B), equating to a 1SD difference.

On the D‐KEFS Tower test, patients scored significantly worse than controls on the Tower Total (*Z* = −3.10, *P* = 0.006, *r* = −0.39), Time Per Move Ratio (*Z* = −3.18, *P* = 0.004, *r* = −0.40), and Rule Violations Per Item Ratio (*Z* = −2.52, *P* = 0.024, *r* = −0.32; Table S2B). There was no significant difference in Move Accuracy Ratio (*Z* = −0.37, *P* = 0.712, *r* = −0.05).

#### Memory

There was no significant difference between patients and matched controls on any WMS‐IV memory subtests (Table S2C).

### Change in patient cognition from premorbid to current levels

Patients declined by 8.37 IQ points on average between the WTAR and WAIS‐IV, a significant change from premorbid to current cognition (*Z*(49) = −4.44, *P* < 0.001, *r* = −0.56). Of these, 37 patients declined between the two measures, 11 increased, and one remained constant; 14 (29%) declined by ≥1SD.

### Change in cognitive performance of patients compared to controls over 18 months

When comparing patients and controls over time, both groups showed a significant effect of time on WAIS‐IV FSIQ (*P* = 0.026), PRI (*P* = 0.044), D‐KEFS Tower Total (*P* = 0.012), and Rule Violations Per Item Ratio (*P* = 0.022); and on WMS‐IV LM I (*P* = 0.021), LM II (*P* = 0.021), LM Recognition (*P* = 0.021), and VPA I (*P* = 0.021). There were no significant interactions (Table S2A‐C).

### Predictors of cognition in patients with mitochondrial disease

Table 3 describes predictors of cognitive ability in patients with mitochondrial disease.

#### Premorbid cognition

WTAR showed a positive relationship with number of years in education, and a negative relationship with NMDAS, and anticonvulsant medication, accounting for 55.7% of the variance in WTAR FSIQ.

#### General cognitive functioning

WTAR FSIQ was a significant, positive predictor of all WAIS‐IV scores. Disease‐specific factors also showed a significant negative relationship with performance on all but WMI. Additionally, BAI was a significant negative predictor of WAIS‐IV FSIQ, the BDI was a significant negative predictor of PRI, and age was a significant positive predictor of WMI. Variance accounted for by the models ranged from 55.2% to 80.2%.

#### Executive function

WTAR FSIQ had a significant positive relationship with all D‐KEFS subtests except VF Letter and Tower Move Accuracy Ratio, while disease‐specific factors showed a significant negative relationship with all D‐KEFS subtests. Anticonvulsant medication showed a significant negative relationship with VF Letter, female gender showed a positive relationship with both VF Switching Correct and Switching Accuracy, and BAI was a significant negative predictor of Tower Time Per Move Ratio. Additionally, there was a significant interaction between WTAR and NMDAS on VF Category; NMDAS had a greater effect on performance as WTAR increased (Fig. S2). A significant interaction between WTAR FSIQ and % mtDNA mutation level in urine on Tower Total showed that WTAR had a significant positive affect on Tower Total at lower % mtDNA mutation levels, but did not affect performance for those with higher % mtDNA mutation levels (Fig. S3). The significant interaction between WTAR FSIQ and mtDNA genotype on Tower Rule Violations Per Item Ratio (using transformed (squared) scores to correct for nonnormality of unstandardized residuals) showed that patients with the m.8344A>G point mutation who had lower premorbid cognitive ability (as assessed by WTAR) made more rule violations than patients with the m.3243A>G mutation, or patients with m.8344A>G and higher premorbid cognitive ability (Fig. S4). Variance accounted for by the models ranged from 27.3% to 75.7%.

#### Memory

WTAR was the most influential predictor of memory in patients. Anticonvulsant medication showed a significant negative relationship with LM I performance and BDI was a significant negative predictor of VPA I and VPA II. Disease‐specific factors significantly predicted performance on only VPA II and VPA Free Recall (Table 3). Variance accounted for by the models ranged from 33.9% to 76.0%.

**Table 3:** Linear models showing predictors of cognitive ability in patients with mitochondrial disease

Test	B (CI)	SE B	*β*	*P*	*R* ^2^
WTAR FSIQ					0.557
(Constant)	75.08 (60.20, 89.97)	7.39		<0.001	
No. of years in education	1.99 (1.00, 2.98)	0.49	0.41	<0.001	
NMDAS	−0.24 (−0.45, −0.03)	0.10	−0.25	0.026	
Anticonvulsant medication	−11.97 (−17.80, −6.13)	2.90	−0.45	<0.001	
WAIS‐IV					
FSIQ					0.802
(Constant)	32.44 (10.09, 54.78)	11.09		0.005	
WTAR FSIQ	0.76 (0.56, 0.97)	0.10	0.58	<0.001	
% mtDNA mutation level in urine	−0.16 (−0.25, −0.07)	0.04	−0.25	0.001	
BAI	−0.56 (−0.80, −0.32)	0.12	−0.36	<0.001	
VCI					0.777
(Constant)	11.92 (−6.82, 30.66)	9.31		0.207	
WTAR FSIQ	0.86 (0.68, 1.04))	0.09	0.78	<0.001	
NMDAS	−0.18 (−0.35, −0.01)	0.08	−0.17	0.039	
PRI					0.552
(Constant)	41.43 (9.00, 73.87)	16.10		0.013	
WTAR FSIQ	0.70 (0.41, 1.00)	0.15	0.51	<0.001	
% mtDNA mutation level in urine	−0.16 (−0.29, −0.02)	0.07	−0.24	0.026	
BDI	−0.34 (−0.61, −0.07)	014	−0.27	0.015	
WMI					0.593
(Constant)	−4.78 (−27.39, 17.84)	11.23		0.673	
WTAR FSIQ	0.82 (0.59, 1.05)	0.11	0.69	<0.001	
Age	0.32 (0.08, 0.55)	0.12	0.26	0.010	
PSI					0.665
(Constant)	58.56 (24.25, 92.88)	17.03		0.001	
WTAR FSIQ	0.55 (0.23, 0.86)	0.16	0.35	0.001	
% mtDNA mutation level in urine	−0.24 (−0.37, −0.11)	0.07	0.34	0.001	
NMDAS	−0.58 (−0.87, −0.29)	0.15	0.40	<0.001	
D‐KEFS					
VF Letter					0.457
(Constant)	11.11 (9.68, 12.53)	0.71		<0.001	
NMDAS	−0.11 (−0.17, −0.05)	0.03	−0.41	0.001	
Anticonvulsant medication	−2.92 (−4.71, −1.14)	0.89	−0.39	0.002	
VF category					0.757
(Constant)	−10.14 (−21.18, 0.91)	5.47		0.071	
WTAR FSIQ	0.23 (0.12, 0.35)	0.06	0.72	<0.001	
% mtDNA mutation level in urine	−0.04 (−0.07, −0.02)	0.01	−0.27	0.002	
NMDAS	0.31 (−0.10, −0.72)	0.20	1.05	0.134	
WTAR FSIQ * NMDAS	−0.01 (−0.01, 0.00)	0.00	−1.39	0.031	
VF switching correct					0.454
(Constant)	−0.97 (−10.30, 8.35)	4.63		0.834	
WTAR FSIQ	0.10 (0.02, 0.19)	0.04	0.30	0.023	
NMDAS	−0.11 (−0.19, −0.02)	0.04	−0.33	0.014	
Gender	3.00 (1.09, 4.92)	0.95	0.35	0.003	
VF switching accuracy					0.446
(Constant)	2.38 (−5.27, 10.03)	3.80		0.534	
WTAR FSIQ	0.08 (0.01, 0.15)	0.04	0.28	0.035	
NMDAS	−0.10 (−0.17, −0.03)	0.03	−0.37	0.006	
Gender	2.16 (0.58, 3.73)	0.78	0.31	0.008	
Tower total					0.444
(Constant)	27.26 (11.27, 43.26)	7.94		0.001	
WTAR FSIQ	−0.17 (−0.33, −0.00)	0.08	−0.72	0.046	
% mtDNA mutation level in urine	−0.40 (−0.63, −0.18)	0.11	−3.62	0.001	
WTAR FSIQ * % mtDNA mutation level in urine	0.00 (0.00, 0.01)	0.00	3.23	0.002	
Tower time per move ratio					0.423
(Constant)	1.52 (−7.03, 10.08)	4.25		0.721	
WTAR FSIQ	0.10 (0.03, 0.18)	0.04	0.34	0.010	
% mtDNA mutation level in urine	−0.04 (−0.08, −0.01)	0.02	−0.30	0.013	
BAI	−0.09 (−0.18, −0.00)	0.04	−0.26	0.042	
Tower move accuracy ratio					0.273
(Constant)	8.03 (6.84, 9.22)	0.59		<0.001	
NMDAS	0.10 (0.05, 0.15)	0.02	0.52	<0.001	
Tower rule violations per item ratio (squared)					0.483
(Constant)	224.16 (51.84, 396.47)	85.56		0.012	
WTAR FSIQ	−1.17 (−3.02, 0.68)	0.92	−0.43	0.209	
mtDNA genotype	−195.09 (−320.62, 69.57)	62.32	−2.55	0.003	
WTAR FSIQ * mtDNA genotype	1.89 (0.50, 3.27)	0.69	2.16	0.009	
WMS‐IV					
LM I					0.445
(Constant)	−4.17 (−11.68, 3.35)	3.73		0.270	
WTAR FSIQ	0.13 (0.06, 0.21)	0.04	0.46	0.001	
Anticonvulsant medication	−2.25 (−4.27, −0.23)	1.00	−0.30	0.030	
LM II					0.339
(Constant)	−6.27 (−12.00, −0.53)	2.85		0.033	
WTAR FSIQ	0.15 (0.09, 0.21)	0.03	0.58	<0.001	
VPA I					0.523
(Constant)	−4.27 (−10.52, 1.97)	3.10		0.175	
WTAR FSIQ	0.16 (0.10, 0.22)	0.03	0.57	<0.001	
BDI	−0.08 (−0.14, −0.02)	0.03	−0.30	0.007	
VPA II					0.475
(Constant)	1.98 (−6.00, 9.97)	3.96		0.619	
WTAR FSIQ	0.13 (0.05, 0.20)	0.04	0.40	0.001	
% mtDNA mutation level in urine	−0.04 (−0.07, −0.00)	0.02	−0.24	0.037	
BDI	−0.09 (−0.16, −0.03)	−0.09	0.03	0.008	
VPA free recall					0.760
(Constant)	−5.24 (−11.02, 0.54)	2.87		0.074	
WTAR FSIQ	0.19 (0.14, 0.24)	0.03	0.62	<0.001	
% mtDNA mutation level in urine	−0.03 (−0.05, −0.01)	0.01	−0.21	0.009	
NMDAS	−0.08 (−0.13, −0.03)	0.03	−0.28	0.003	
Change in cognition from premorbid to baseline levels					
WTAR‐WAIS‐IV change					0.424
(Constant)	7.43 (1.10, 13.76)	3.14		0.022	
% mtDNA mutation level in urine	−0.16 (−0.25, −0.07)	0.05		0.001	
NMDAS	−0.32 (−0.51, −0.13_	0.09		0.001	

Abbreviations: WTAR FSIQ: Wechsler test of adult reading; WAIS‐IV: Wechsler adult intelligence scale‐IV; FSIQ: FSIQ; VCI: verbal comprehension index; PRI: perceptual reasoning index; WMI: working memory index; PSI: processing speed index; D‐KEFS VF: Delis‐Kaplan executive function score verbal fluency; LM: logical memory; VPA: verbal paired associates; BDI: Beck's depression inventory; BAI: Beck's anxiety inventory; NMDAS: Newcastle mitochondrial disease adult scale.

#### Change in cognition from premorbid to baseline levels

Change in cognition from premorbid estimates to baseline levels had a significant negative relationship with % mtDNA mutation level in urine and NMDAS and accounted for 42.4% of the variance (Fig. S5).

## Discussion

We sought to systematically investigate cognition in a well‐defined cohort of patients with m.3243A>G and m.8344A>G‐related mitochondrial disease. Following identification of deficits compared to normative data, we compared patients to control participants matched by premorbid cognitive ability, in an attempt to define the effect of baseline cognition. This study showed mild‐to‐moderate premorbid cognitive difficulties, that is, the group distribution was lower overall than the national expectations for intellectual attainment. Patients also showed substantial impairment in current functioning in the domains of verbal comprehension, perceptual reasoning, working memory, and processing speed (after correction for motor speed difficulties). Compared to matched controls, patients performed worse on all domains, except memory and executive function. With regards to memory, patients did demonstrate substantial memory retrieval difficulties; however, this did not vary significantly from our control population, who had the same level of baseline IQ.

High rates of general and specific cognitive impairment, compared to the norm and to matched controls, were identified, broadly corroborating previous literature.^2^, ^3^, ^4^, ^6^, ^7^, ^8^, ^9^, ^23^ We identified relative strengths in perceptual reasoning compared to processing speed. Although a seven point difference between two cognitive domains (PRI and PSI), is not rare within the general population,^13^ 37% of the sample showed a clinically significant difference (>1SD difference). Executive function performance was more variable and impairments may potentially be caused by other difficulties (e.g. word generation; slow but accurate decision‐making). These findings are in contrast with other studies, that report clear evidence of executive dysfunction.^2^, ^3^, ^6^, ^11^ Indeed, our results would suggest that these deficits, in part, may result from slower decision‐making.

This study also found that, although immediate and delayed free recall were significantly worse than the norm, heightened recognition performance demonstrated difficulties with memory retrieval, rather than encoding or storage. Furthermore, patients did not differ from controls on any of the WMS‐IV memory tests, indicating that while memory problems do exist, those difficulties are no worse than would be expected based on premorbid cognitive ability. Previous studies interrogating memory have demonstrated inconsistencies^2^, ^3^, ^6^, ^9^, ^10^, ^11^ while this study clearly identifies memory retrieval as the impaired step in this process.

Patients with m.3243A>G and m.8344A>G‐related mitochondrial disease showed cognitive decline from premorbid estimates to current levels, and while mean decline was eight IQ points, 28% showed declines of >1SD. Despite this suggestion of longer term decline, patients showed the same pattern of cognitive improvements as controls over the length of this study, in line with practice effects caused by familiarity, and indicative of normal learning ability. However, given that premorbid cognitive ability of matched pairs was within the normal range, conclusions can only be drawn about patients within this range. It cannot be inferred whether the same pattern of results would be found for those with lower premorbid cognition (and worse disease profile), who might show differential practice effects or sharper cognitive declines over time. While this group analysis does not allow discussion of individual variability, using our robust cognitive battery, this may help delineate decline in specific domains in more severely affected patient populations. In this sample, the contrasting results indicating both decline and stability may suggest slow changes over a longer period than is measured here^7^ and/or sudden declines linked to discrete changes in other disease‐specific factors. In keeping with this, disease severity, measured by the NMDAS, remained stable over the 18‐month follow‐up period (Table S2D); thus, disease‐specific factors that might impact cognitive change were unlikely to trigger cognitive decline. Importantly, with 15% patient attrition at 18 months, caused by general health deterioration, a different pattern may have emerged had this subsample remained. It is important to recognize the possibility of individual variability in day‐to‐day functioning in patients with mitochondrial disease,^24^, ^25^, ^26^, ^27^ which could impact on cognitive performance each time participants are assessed. However, long‐term, regular observation would be necessary to identify disease‐related within person variability in cognition, and still, practice effects would make it challenging to capture this variability.

Premorbid cognitive ability was a significant predictor of many areas of cognition, as seen in normal populations.^28^, ^29^ More interestingly, we found a significant negative relationship between disease severity and some measures of current cognition, as opposed to genotypic differences identified previously,^23^ and change in cognition (WTAR‐WAIS‐IV) was predicted solely by disease severity, that was not unduly impacted by central nervous system involvement, as assessed by NMDAS. Indeed, mtDNA genotype only predicted that patients harboring the m.8344A>G mutation who had lower premorbid cognition made more rule violations on the Tower test. However, caution is required when interpreting differences caused by mtDNA genotype, given the small numbers of m.8344A>G patients. Additionally, anticonvulsant medication and mood negatively predicted different cognitive abilities. While our sample size is small and allowed little power for these analyses (requiring validation with larger samples), it is the largest systematic investigation of distinct cognitive domains in patients to date.^2^, ^3^, ^4^, ^5^, ^6^, ^7^, ^8^, ^9^, ^10^, ^11^, ^23^

An inherent limitation is that while WTAR is a reliable tool for estimating peak cognition before onset of decline caused by illness/injury^30^, ^31^; reading tests are not completely impervious to cerebral insult,^32^, ^33^, ^34^ and it is unknown how cerebral damage affects reading ability in patients. However, mixed effect modeling using WTAR data from the MRC Mitochondrial Disease Cohort UK (576 observations from 157 patients harboring either the m.3243A>G or m.8344A>G mutation, with two or more WTAR scores) showed that, after accounting for between‐patient variability, there was no significant change in WTAR score over time (*P* > 0.05). This confirms its stability over multiple assessments and supports use of the WTAR as an analogue for premorbid cognition in this patient cohort.

## Conclusions

These findings have important implications for clinical practice and for the advice provided to ameliorate everyday difficulties when cognitive problems present in this subset of patients. Early educational support, to ensure that children with mitochondrial disease achieve their full potential, may provide protective effects against later cognitive difficulties. Indications of slow cognitive decline suggest that patients should be monitored over time to identify difficulties early, and if cognitive difficulties do develop, appropriate compensatory strategies should be identified through in‐depth cognitive assessment. When considering clinical trials, this in‐depth cognitive profile would also be invaluable for assessing the impact of any potential therapies. In clinical practice, healthcare professionals should be alert to disease‐specific factors, which may increase the likelihood of cognitive difficulties, and monitor those individuals more closely. Healthcare professionals should also be mindful of how cognitive difficulties may influence understanding of information and subsequent ability to provide informed consent, and consider how this risk might be mitigated. Practical steps that can be taken in everyday life, as well as in clinical settings, could include using strategies such as slower presentation of information; multiple modes of presentation to reinforce information and exploit relative cognitive strengths; active discussion to aid understanding and decision‐making; and memory aids, including repetition and cues.

## Author Contributions

HM: Conception and design of the study; acquisition and analysis of data; drafting the manuscript and figures.

TK: Conception and design of the study; drafting and revision of the manuscript.

AB: Acquisition of data; drafting and revision of the manuscript.

RHF: Analysis of data; drafting and revision of the manuscript.

AMS: Acquisition of data; drafting and revision of the manuscript.

APB: Analysis of data; drafting and revision of the manuscript.

RWT: Acquisition and analysis of data; drafting the manuscript and figures.

RMF: Conception and design of the study; drafting and revision of the manuscript.

DMT: Conception and design of the study; drafting and revision of the manuscript.

GSG: Conception and design of the study; acquisition and analysis of data; drafting the manuscript and figures.

## Conflict of Interest

None declared.

## Supporting information


**Figure S1.** WAIS‐IV PSI performance, controlling for motor speed.
**Figure S2.** Relationship between WTAR FSIQ and D‐KEFS VF Category, grouped by NMDAS level. NMDAS was split into two groups using the mean of 20.
**Figure S3.** Relationship between WTAR FSIQ and D‐KEFS Tower Total, grouped by % mtDNA mutation level in urine. % mtDNA mutation level in urine was split into two groups using the mean of 60.
**Figure S4.** Relationship between WTAR FSIQ and D‐KEFS Tower Rule Violations Per Item Ratio (squared), grouped by mtDNA genotype.
**Figure S5.** Relationship between % mtDNA mutation level in urine and NMDAS on change in cognition, from premorbid estimates to baseline levels. NMDAS was split into two groups using the mean of 20.

**Table S1.** Patient cognitive performance compared to normative data.

**Table S2.** (A) Patient WAIS‐IV performance over time and in comparison to matched controls. (B) Patient D‐KEFS performance over time and in comparison to matched controls. (C) Patient WMS‐IV performance over time and in comparison to matched controls. (D) Patient NMDAS scores over time.

**Data S1.** Description of validation samples for each cognitive assessment, and in depth description statistical analyses.
