# Supplementary material for: Cognitive deficits in adult m.3243A>G‐ and m.8344A>G‐related mitochondrial disease: importance of correcting for baseline intellectual ability
Source: Ann Clin Transl Neurol. 2019 Mar 27;6(5):826–36. doi: 10.1002/acn3.736 (PMC6529924; doi:10.1002/acn3.736)
Supplement: Supplementary file 1 — Figure S1. WAIS‐IV PSI performance, controlling for motor speed. Figure S2. Relationship between WTAR FSIQ and D‐KEFS VF Category, grouped by NMDAS level. NMDAS was split into two groups using the mean of 20. Figure S3. Relationship between WTAR FSIQ and D‐KEFS Tower Total, grouped by % mtDNA mutation level in urine. % mtDNA mutation level in urine was split into two groups using the mean of 60. Figure S4. Relationship between WTAR FSIQ and D‐KEFS Tower Rule Violations Per Item Ratio (squared), grouped by mtDNA genotype. Figure S5. Relationship between % mtDNA mutation level in urine and NMDAS on change in cognition, from premorbid estimates to baseline levels. NMDAS was split into two groups using the mean of 20. [file ACN3-6-826-s001.docx]

Supplementary Figures

Supplementary Figure 1. WAIS-IV PSI performance, controlling for motor speed.

Supplementary Figure 2. Relationship between WTAR FSIQ and D-KEFS VF Category, grouped by NMDAS level. NMDAS was split into two groups using the mean of 20.

Supplementary Figure 3. Relationship between WTAR FSIQ and D-KEFS Tower Total, grouped by % mtDNA mutation level in urine. % mtDNA mutation level in urine was split into two groups using the mean of 60.

Supplementary Figure 4. Relationship between WTAR FSIQ and D-KEFS Tower Rule Violations Per Item Ratio (squared), grouped by mtDNA genotype.

Supplementary Figure 5. Relationship between % mtDNA mutation level in urine and NMDAS on change in cognition, from premorbid estimates to baseline levels. NMDAS was split into two groups using the mean of 20.
