# Supplementary material for: Cognitive deficits in adult m.3243A>G‐ and m.8344A>G‐related mitochondrial disease: importance of correcting for baseline intellectual ability
Source: Ann Clin Transl Neurol. 2019 Mar 27;6(5):826–36. doi: 10.1002/acn3.736 (PMC6529924; doi:10.1002/acn3.736)
Supplement: Supplementary file 2 — Table S1. Patient cognitive performance compared to normative data. [file ACN3-6-826-s002.docx]

Supplementary Table 1. Patient cognitive performance compared to normative data.

| Test | Mean | SD | p | d |
| --- | --- | --- | --- | --- |
| WTAR (M:100 (15)) | | | | |
| FSIQ | 93.45 | 12.59 | .002* | -0.47 |
| WAIS-IV (M: 100 (15)) | | | | |
| FSIQ | 85.08 | 17.42 | .005* | -0.92 |
| VCI | 88.76 | 13.85 | .005* | -0.78 |
| PRI | 91.41 | 17.36 | .005* | -0.53 |
| WMI | 86.37 | 14.91 | .005* | -0.91 |
| PSI | 84.04 | 19.29 | .005* | -0.92 |
| D-KEFS (M: 10 (3)) | | | | |
| VF Letter | 7.88 | 3.53 | .008* | -0.65 |
| VF Category | 7.12 | 4.28 | .008* | -0.78 |
| VF Switching Correct | 8.02 | 4.33 | .008* | -0.53 |
| VF Switching Accuracy | 8.8 | 3.53 | .015* | -0.37 |
| Tower Total | 8.61 | 2.94 | .008* | -0.47 |
| Tower Time Per Move Ratio | 7.22 | 3.81 | .008* | -0.81 |
| Tower Move Accuracy Ratio | 9.96 | 2.74 | .924 | -0.01 |
| Tower Rule Violations Per Item Ratio | 9.08 | 2.38 | .064 | -0.34 |
| WMS-IV (M: 10 (3)) | | | | |
| LM I | 7.31 | 3.60 | .005* | -0.81 |
| LM II | 7.59 | 3.21 | .005* | -0.77 |
| VPA I | 9.43 | 3.60 | .364 | -0.17 |
| VPA II | 9.94 | 3.95 | .886 | -0.02 |
| VPA Free Recall | 8.84 | 3.92 | .021* | -0.33 |

*: Significant
