# Supplementary material for: Cognitive deficits in adult m.3243A>G‐ and m.8344A>G‐related mitochondrial disease: importance of correcting for baseline intellectual ability
Source: Ann Clin Transl Neurol. 2019 Mar 27;6(5):826–36. doi: 10.1002/acn3.736 (PMC6529924; doi:10.1002/acn3.736)
Supplement: Supplementary file 3 — Table S2. (A) Patient WAIS‐IV performance over time and in comparison to matched controls. (B) Patient D‐KEFS performance over time and in comparison to matched controls. (C) Patient WMS‐IV performance over time and in comparison to matched controls. (D) Patient NMDAS scores over time. [file ACN3-6-826-s003.docx]

Supplementary Table 2a. Patient WAIS-IV performance over time and in comparison to matched controls.

| Test | Time | Group | Mean | SD | p (Disease) | p (Time) | p (Interaction) |
| --- | --- | --- | --- | --- | --- | --- | --- |
| Patient-Control Comparison of WAIS-IV at Baseline Assessment | | | | | | | |
| FSIQ | 1 | P | 92.69 | 14.48 | .005* |  |  |
|  |  | C | 109.66 | 13.26 |  |  |  |
| VCI | 1 | P | 95.72 | 10.74 | .005* |  |  |
|  |  | C | 107.53 | 13.41 |  |  |  |
| PRI | 1 | P | 97.28 | 15.95 | .005* |  |  |
|  |  | C | 110.06 | 14.09 |  |  |  |
| WMI | 1 | P | 92.56 | 12.78 | .005* |  |  |
|  |  | C | 102.81 | 14.70 |  |  |  |
| PSI | 1 | P | 89.72 | 17.18 | .005* |  |  |
|  |  | C | 108.34 | 12.18 |  |  |  |
| Change in WAIS-IV Performance of Patients over 18 Months | | | | | | | |
| FSIQ | 1 | P | 87.12 | 17.54 |  | .003* |  |
|  | 2 |  | 89.67 | 19.30 |  |  |  |
|  | 3 |  | 89.20 | 19.76 |  |  |  |
| VCI | 1 | P | 89.43 | 14.55 |  | .066 |  |
|  | 2 |  | 91.98 | 16.05 |  |  |  |
|  | 3 |  | 90.31 | 16.64 |  |  |  |
| PRI | 1 | P | 93.40 | 17.74 |  | .248 |  |
|  | 2 |  | 94.83 | 19.52 |  |  |  |
|  | 3 |  | 95.48 | 18.18 |  |  |  |
| WMI | 1 | P | 87.61 | 15.15 |  | .248 |  |
|  | 2 |  | 89.64 | 15.59 |  |  |  |
|  | 3 |  | 88.10 | 15.10 |  |  |  |
| PSI | 1 | P | 86.50 | 18.92 |  | .003* |  |
|  | 2 |  | 89.26 | 20.99 |  |  |  |
|  | 3 |  | 90.12 | 21.10 |  |  |  |
| Change in WAIS-IV Performance of Patients over 18 Months Compared to Matched Controls | | | | | | | |
| FSIQ | 1 | P | 98.35 | 13.99 | .015* | .026* | 1.000 |
|  |  | C | 113.75 | 11.41 |  |  |  |
|  | 2 | P | 102.20 | 15.87 |  |  |  |
|  |  | C | 116.20 | 12.94 |  |  |  |
|  | 3 | P | 100.70 | 16.75 |  |  |  |
|  |  | C | 117.05 | 13.98 |  |  |  |
| VCI | 1 | P | 99.05 | 10.71 | .14 | 1.000 | 1.000 |
|  |  | C | 109.95 | 12.53 |  |  |  |
|  | 2 | P | 102.05 | 12.79 |  |  |  |
|  |  | C | 110.15 | 12.09 |  |  |  |
|  | 3 | P | 100.80 | 12.35 |  |  |  |
|  |  | C | 111.20 | 15.85 |  |  |  |
| PRI | 1 | P | 103.30 | 16.12 | .272 | .044* | 1.000 |
|  |  | C | 113.75 | 9.39 |  |  |  |
|  | 2 | P | 108.15 | 17.10 |  |  |  |
|  |  | C | 117.30 | 13.42 |  |  |  |
|  | 3 | P | 105.20 | 17.39 |  |  |  |
|  |  | C | 114.65 | 12.46 |  |  |  |
| WMI | 1 | P | 96.00 | 13.34 | .015* | .576 | .272 |
|  |  | C | 105.50 | 13.56 |  |  |  |
|  | 2 | P | 97.50 | 14.10 |  |  |  |
|  |  | C | 110.00 | 14.33 |  |  |  |
|  | 3 | P | 94.25 | 14.62 |  |  |  |
|  |  | C | 114.80 | 15.55 |  |  |  |
| PSI | 1 | P | 94.60 | 17.90 | .036* | .270 | 1.000 |
|  |  | C | 112.45 | 12.48 |  |  |  |
|  | 2 | P | 97.85 | 19.97 |  |  |  |
|  |  | C | 112.75 | 11.63 |  |  |  |
|  | 3 | P | 99.15 | 19.61 |  |  |  |
|  |  | C | 113.75 | 14.44 |  |  |  |

*: Significant

Supplementary Table 2b. Patient D-KEFS performance over time and in comparison to matched controls.

| Test | Time | Group | Mean | SD | p (Disease) | p (Time) | p (Interaction) |
| --- | --- | --- | --- | --- | --- | --- | --- |
| Patient-Control Comparison of D-KEFS at Baseline Assessment | | | | | | | |
| *Verbal Fluency Subtests* | | | | | | | |
| Letter | 1 | P | 9.53 | 3.45 | .004* |  |  |
|  |  | C | 12.75 | 3.03 |  |  |  |
| Category | 1 | P | 8.63 | 3.87 | .004* |  |  |
|  |  | C | 12.47 | 3.70 |  |  |  |
| Switching Correct | 1 | P | 9.50 | 3.60 | .004* |  |  |
|  |  | C | 12.69 | 3.38 |  |  |  |
| Switching Accuracy | 1 | P | 10.03 | 3.02 | .004* |  |  |
|  |  | C | 12.97 | 2.92 |  |  |  |
| *Tower Subtests* | | | | | | | |
| Total | 1 | P | 9.31 | 2.39 | .006* |  |  |
|  |  | C | 11.72 | 2.61 |  |  |  |
| Time Per Move Ratio | 1 | P | 8.25 | 3.36 | .004* |  |  |
|  |  | C | 10.91 | 2.07 |  |  |  |
| Move Accuracy Ratio | 1 | P | 9.72 | 2.68 | .712 |  |  |
|  |  | C | 9.41 | 2.12 |  |  |  |
| Rule Violations Per Item Ratio | 1 | P | 9.59 | 2.05 | .024* |  |  |
|  |  | C | 10.53 | 0.80 |  |  |  |
| Change in D-KEFS Performance of Patients over 18 Months | | | | | | | |
| *Verbal Fluency Subtests* | | | | | | | |
| Letter | 1 | P | 8.14 | 3.55 |  | .246 |  |
|  | 2 |  | 8.21 | 4.08 |  |  |  |
|  | 3 |  | 9.00 | 4.50 |  |  |  |
| Category | 1 | P | 7.43 | 4.43 |  | .112 |  |
|  | 2 |  | 8.07 | 4.96 |  |  |  |
|  | 3 |  | 8.43 | 5.00 |  |  |  |
| Switching Correct | 1 | P | 8.40 | 4.45 |  | .246 |  |
|  | 2 |  | 8.60 | 4.83 |  |  |  |
|  | 3 |  | 9.60 | 5.41 |  |  |  |
| Switching Accuracy | 1 | P | 9.10 | 3.61 |  | .254 |  |
|  | 2 |  | 9.31 | 4.41 |  |  |  |
|  | 3 |  | 9.98 | 5.08 |  |  |  |
| *Tower Subtests* | | | | | | | |
| Total | 1 | P | 8.93 | 2.88 |  | .004* |  |
|  | 2 |  | 10.45 | 3.09 |  |  |  |
|  | 3 |  | 11.90 | 3.18 |  |  |  |
| Timer Per Move Ratio | 1 | P | 7.48 | 3.72 |  | .004* |  |
|  | 2 |  | 9.02 | 3.27 |  |  |  |
|  | 3 |  | 9.45 | 3.07 |  |  |  |
| Move Accuracy Ratio | 1 | P | 9.74 | 2.65 |  | .994 |  |
|  | 2 |  | 9.79 | 2.01 |  |  |  |
|  | 3 |  | 9.76 | 2.96 |  |  |  |
| Rule Violations Per Item Ratio | 1 | P | 9.33 | 2.31 |  | .004* |  |
|  | 2 |  | 10.19 | 1.52 |  |  |  |
|  | 3 |  | 10.55 | 1.06 |  |  |  |
| Change in D-KEFS Performance of Patients over 18 Months Compared to Matched Controls | | | | | | | |
| *Verbal Fluency Subtests* | | | | | | | |
| Letter | 1 | P | 9.30 | 3.45 | .066 | 1.000 | .994 |
|  |  | C | 13.35 | 3.45 |  |  |  |
|  | 2 | P | 9.65 | 3.80 |  |  |  |
|  |  | C | 13.55 | 3.95 |  |  |  |
|  | 3 | P | 10.55 | 4.84 |  |  |  |
|  |  | C | 12.90 | 4.38 |  |  |  |
| Category | 1 | P | 9.65 | 4.22 | .060 | 1.000 | 1.000 |
|  |  | C | 13.55 | 3.44 |  |  |  |
|  | 2 | P | 10.10 | 5.03 |  |  |  |
|  |  | C | 13.70 | 3.57 |  |  |  |
|  | 3 | P | 10.25 | 4.64 |  |  |  |
|  |  | C | 13.15 | 3.50 |  |  |  |
| Switching Correct | 1 | P | 9.85 | 3.18 | .150 | .504 | 1.000 |
|  |  | C | 12.85 | 3.69 |  |  |  |
|  | 2 | P | 10.20 | 3.81 |  |  |  |
|  |  | C | 13.45 | 3.10 |  |  |  |
|  | 3 | P | 11.70 | 4.64 |  |  |  |
|  |  | C | 13.55 | 4.33 |  |  |  |
| Switching Accuracy | 1 | P | 10.40 | 2.80 | .153 | 1.000 | 1.000 |
|  |  | C | 13.35 | 3.03 |  |  |  |
|  | 2 | P | 11.00 | 3.29 |  |  |  |
|  |  | C | 13.40 | 2.48 |  |  |  |
|  | 3 | P | 11.90 | 4.28 |  |  |  |
|  |  | C | 13.50 | 4.43 |  |  |  |
| *Tower Subtests* | | | | | | | |
| Total | 1 | P | 9.55 | 2.93 | .700 | .012* | 1.000 |
|  |  | C | 11.75 | 2.59 |  |  |  |
|  | 2 | P | 11.65 | 3.50 |  |  |  |
|  |  | C | 12.50 | 2.84 |  |  |  |
|  | 3 | P | 12.30 | 3.26 |  |  |  |
|  |  | C | 13.40 | 2.64 |  |  |  |
| Time Per Move Ratio | 1 | P | 8.80 | 3.19 | .342 | .06 | 1.000 |
|  |  | C | 11.00 | 2.25 |  |  |  |
|  | 2 | P | 10.15 | 3.01 |  |  |  |
|  |  | C | 11.05 | 2.54 |  |  |  |
|  | 3 | P | 10.20 | 2.71 |  |  |  |
|  |  | C | 11.80 | 1.28 |  |  |  |
| Move Accuracy Ratio | 1 | P | 9.70 | 2.30 | 1.000 | 1.000 | 1.000 |
|  |  | C | 9.70 | 2.11 |  |  |  |
|  | 2 | P | 10.25 | 1.68 |  |  |  |
|  |  | C | 9.70 | 3.21 |  |  |  |
|  | 3 | P | 9.55 | 3.71 |  |  |  |
|  |  | C | 10.30 | 2.60 |  |  |  |
| Rule Violations Per Item Ratio | 1 | P | 9.85 | 2.06 | 1.000 | .022* | .504 |
|  |  | C | 10.70 | 0.47 |  |  |  |
|  | 2 | P | 10.55 | 1.57 |  |  |  |
|  |  | C | 10.85 | 0.37 |  |  |  |
|  | 3 | P | 10.55 | 1.36 |  |  |  |
|  |  | C | 10.85 | 0.37 |  |  |  |

*: Significant

Supplementary Table 2c. Patient WMS-IV performance over time and in comparison to matched controls.

| Test | Time | Group | Mean | SD | p (Disease) | p (Time) | p (Interaction) |
| --- | --- | --- | --- | --- | --- | --- | --- |
| Patient-Control Comparison at Baseline Assessment | | | | | | | |
| LM I | 1 | P | 8.69 | 3.07 | .300 |  |  |
|  |  | C | 9.88 | 3.03 |  |  |  |
| LM II | 1 | P | 8.66 | 2.94 | .295 |  |  |
|  |  | C | 10.09 | 3.25 |  |  |  |
| VPA I | 1 | P | 10.75 | 3.08 | .300 |  |  |
|  |  | C | 12.25 | 3.79 |  |  |  |
| VPA II | 1 | P | 11.09 | 3.37 | .839 |  |  |
|  |  | C | 11.41 | 2.96 |  |  |  |
| VPA Free Recall | 1 | P | 10.16 | 3.32 | .295 |  |  |
|  |  | C | 11.84 | 3.89 |  |  |  |
| Change in Memory Ability of Patients over 18 Months | | | | | | | |
| LM I | 1 | P | 7.57 | 3.61 |  | .007* |  |
|  | 2 |  | 8.90 | 3.63 |  |  |  |
|  | 3 |  | 9.86 | 3.69 |  |  |  |
| LM II | 1 | P | 7.95 | 2.97 |  | .007* |  |
|  | 2 |  | 9.36 | 3.43 |  |  |  |
|  | 3 |  | 10.31 | 3.78 |  |  |  |
| LM Recognition | 1 | P | 23.60 | 3.21 |  | .007* |  |
|  | 2 |  | 24.43 | 3.44 |  |  |  |
|  | 3 |  | 25.31 | 3.27 |  |  |  |
| VPA I | 1 | P | 9.86 | 3.39 |  | .007* |  |
|  | 2 |  | 11.10 | 3.87 |  |  |  |
|  | 3 |  | 11.48 | 4.03 |  |  |  |
| VPA II | 1 | P | 10.50 | 3.78 |  | .692 |  |
|  | 2 |  | 10.88 | 3.82 |  |  |  |
|  | 3 |  | 10.71 | 3.68 |  |  |  |
| VPA Recognition | 1 | P | 37.05 | 5.23 |  | .692 |  |
|  | 2 |  | 37.88 | 3.76 |  |  |  |
|  | 3 |  | 37.62 | 4.08 |  |  |  |
| VPA Free Recall | 1 | P | 9.24 | 3.75 |  | .007* |  |
|  | 2 |  | 10.62 | 4.10 |  |  |  |
|  | 3 |  | 10.62 | 3.90 |  |  |  |
| Change in Memory Ability of Patients over 18 Months Compared to Matched Controls | | | | | | | |
| LM I | 1 | P | 9.55 | 3.38 | .945 | .021* | .945 |
|  |  | C | 10.40 | 2.91 |  |  |  |
|  | 2 | P | 10.85 | 2.76 |  |  |  |
|  |  | C | 12.05 | 2.65 |  |  |  |
|  | 3 | P | 11.75 | 2.55 |  |  |  |
|  |  | C | 14.30 | 2.74 |  |  |  |
| LM II | 1 | P | 9.50 | 2.84 | .880 | .021* | 1.000 |
|  |  | C | 10.55 | 3.19 |  |  |  |
|  | 2 | P | 11.05 | 2.82 |  |  |  |
|  |  | C | 12.10 | 3.46 |  |  |  |
|  | 3 | P | 12.10 | 2.97 |  |  |  |
|  |  | C | 14.05 | 3.25 |  |  |  |
| LM Recognition | 1 | P | 24.90 | 3.04 | 1.000 | .021* | 1.000 |
|  |  | C | 25.85 | 2.25 |  |  |  |
|  | 2 | P | 25.65 | 2.68 |  |  |  |
|  |  | C | 26.80 | 2.28 |  |  |  |
|  | 3 | P | 26.65 | 2.94 |  |  |  |
|  |  | C | 27.50 | 1.99 |  |  |  |
| VPA I | 1 | P | 11.75 | 2.67 | 1.000 | .021* | 1.000 |
|  |  | C | 13.30 | 3.47 |  |  |  |
|  | 2 | P | 13.20 | 3.25 |  |  |  |
|  |  | C | 14.10 | 3.28 |  |  |  |
|  | 3 | P | 13.55 | 3.43 |  |  |  |
|  |  | C | 14.50 | 2.95 |  |  |  |
| VPA II | 1 | P | 12.25 | 3.01 | 1.000 | 1.000 | 1.000 |
|  |  | C | 11.95 | 2.67 |  |  |  |
|  | 2 | P | 12.75 | 3.48 |  |  |  |
|  |  | C | 12.55 | 2.39 |  |  |  |
|  | 3 | P | 12.35 | 2.81 |  |  |  |
|  |  | C | 12.90 | 2.05 |  |  |  |
| VPA Recognition | 1 | P | 38.85 | 4.03 | 1.000 | 1.000 | 1.000 |
|  |  | C | 39.40 | 1.27 |  |  |  |
|  | 2 | P | 39.35 | 1.87 |  |  |  |
|  |  | C | 39.65 | 0.75 |  |  |  |
|  | 3 | P | 39.05 | 2.21 |  |  |  |
|  |  | C | 39.65 | 0.67 |  |  |  |
| VPA Free Recall | 1 | P | 11.00 | 3.31 | 1.000 | .629 | 1.000 |
|  |  | C | 12.85 | 3.30 |  |  |  |
|  | 2 | P | 12.65 | 3.41 |  |  |  |
|  |  | C | 13.35 | 3.15 |  |  |  |
|  | 3 | P | 11.80 | 3.49 |  |  |  |
|  |  | C | 13.15 | 3.27 |  |  |  |

*: Significant

Supplementary Table 2d. Patient NMDAS scores over time.

| Test | Time | Group | Mean | SD | p (Disease) | p (Time) | p (Interaction) |
| --- | --- | --- | --- | --- | --- | --- | --- |
| Change in NMDAS Score of Patients over 18 Months | | | | | | | |
| NMDAS | 1 | P | 22.24 | 14.28 |  | .409 |  |
|  | 2 |  | 21.36 | 14.52 |  |  |  |
|  | 3 |  | 20.71 | 13.73 |  |  |  |
