# Supplementary material for: Cognitive deficits in adult m.3243A>G‐ and m.8344A>G‐related mitochondrial disease: importance of correcting for baseline intellectual ability
Source: Ann Clin Transl Neurol. 2019 Mar 27;6(5):826–36. doi: 10.1002/acn3.736 (PMC6529924; doi:10.1002/acn3.736)
Supplement: Supplementary file 4 — Data S1. Description of validation samples for each cognitive assessment, and in depth description statistical analyses. [file ACN3-6-826-s004.docx]

**Supplementary File 1**

**Participants**

The Wechsler Test of Adult Reading (WTAR)^1^ was administered to a subset of the UK Wechsler Adult Intelligence Scale-III validation sample and stratified against UK population norms. The validation sample was 331 people between 16 and 80 years. The Wechsler Adult Intelligence Scale-IV (WAIS-IV)^2^ UK validation sample consisted of 270 people (142 females, 128 males) ranging from 16 to 89 years. The population was matched to the demographic characteristics of the UK population from the 2001 Census. The Wechsler Memory Scale-IV (WMS-IV)^3^ sample for UK population was co-normed with WAIS-IV and consisted of 235 people aged 16 to 89 years. As with the WAIS-IV, the population was matched to the demographic characteristics of the UK population from the 2001 Census. The standardisation of the Delis-Kaplan Executive Function Score (D-KEFS)^4^ is 1700 child and adults aged 8 to 89, stratified to matched demographic characteristics of a US population.

**Statistical Analyses**

To assess patient cognition compared to the normative population, Z-tests were executed in Minitab Version 16^5^. Percentage of patients with scores categorised as impaired was also examined. 1SD was defined as mild impairment and 2SD as severe impairment. In a normal population, only 16% fall more than 1SD below the norm and only 2% fall more than 2SD below the norm. An ANCOVA was also conducted to determine the patients with mitochondrial disease evidenced processing speed problems, when accounting for motor speed. Subscores within each of WAIS-IV, D-KEFS and WMS-IV were compared using paired samples t-test and Wilcoxon signed ranks test for paired samples, performed in SPSS Version 19^6^, to more fully elucidate the profile of cognition. A Wilcoxon Signed Ranks Test for paired samples was also performed in SPSS to determine whether there was a decline in cognition from WTAR estimates of FSIQ to current WAIS-IV FSIQ scores. Paired samples t-tests and Wilcoxon signed ranks test for paired samples were performed in SPSS to compare cognitive performance at baseline of patients with mitochondrial disease and matched control participants. Repeated measures ANOVA was employed to determine whether cognition changed over 18 months (data presented in Supplementary Table 1). Twenty matched pairs completed three rounds of testing and their performance over 18 months was compared using a mixed model ANOVA in SPSS Version 19. Linear regression was executed in SPSS Version 19 to determine predictors of cognition in patients with mitochondrial disease at baseline assessment.

**References**

1. Wechsler D. *Wechsler Test of Adult Reading.* San Antonio, TX: Pearson; 2001.

2. Wechsler D. *Wechsler Adult Intelligence Scale-Fourth Edition.* San Antonio, TX: Pearson; 2008.

3. Wechsler D. *Wechsler Memory Scale-Fourth Edition.* San Antonio, TX: Pearson; 2009.

4. Delis DC, Kaplan E, Kramer JH. *Delis-Kaplan executive function system.* San Antonio: The psychological Cooperation a Haarcourt Assessment Company; 2001.

5. *Minitab 16 Statistical Software* [computer program]. State College, PA: Minitab Inc.; 2010.

6. *IBM SPSS Statistics for Windows, Version 19.0.* [computer program]. Armonk, NY: IBM Corp; 2010.
